# MicroRNA regulation of AMPK in nonalcoholic fatty liver disease

**DOI:** 10.1038/s12276-023-01072-3

**Published:** 2023-09-01

**Authors:** Hao Sun, Jongsook Kim Kemper

**Affiliations:** Department of Molecular and Integrative Physiology, School of Molecular and Cellular Biology, University of Illinois at Urbana-Champaign, Urbana, IL 61801 USA

**Keywords:** Obesity, miRNAs

## Abstract

Obesity-associated nonalcoholic fatty liver disease (NAFLD) is the most common chronic liver disease and is the leading cause of liver failure and death. The function of AMP-activated protein kinase (AMPK), a master energy sensor, is aberrantly reduced in NAFLD, but the underlying mechanisms are not fully understood. Increasing evidence indicates that aberrantly expressed microRNAs (miRs) are associated with impaired AMPK function in obesity and NAFLD. In this review, we discuss the emerging evidence that miRs have a role in reducing AMPK activity in NAFLD and nonalcoholic steatohepatitis (NASH), a severe form of NAFLD. We also discuss the underlying mechanisms of the aberrant expression of *miRs* that can negatively impact AMPK, as well as the therapeutic potential of targeting the miR-AMPK pathway for NAFLD/NASH.

## Liver disease: Aberrant microRNAs impair function of key metabolic protein

MicroRNAs that regulate the activity of a master energy-sensing protein in the cell offer promising therapeutic targets for the treatment of chronic liver disease. Hao Sun and Jongsook Kim Kemper from the University of Illinois at Urbana-Champaign, USA, review how aberrantly expressed microRNAs contribute to the impaired function of AMP-activated protein kinase (AMPK), an energy-sensing protein crucial for various metabolic processes in the liver. Such microRNAs directly target AMPK subunits and indirectly affect signaling pathways. In conditions associated with obesity, imbalanced microRNA expression leads to reduced AMPK activity, resulting in fat accumulation in the liver, impaired glucose metabolism, increased inflammation, and oxidative stress. Restoring normal microRNA levels through drugs holds potential for reversing the symptoms of nonalcoholic fatty liver disease, which can progress to the severe disease known as nonalcoholic steatohepatitis.

## Introduction

Nonalcoholic fatty liver disease (NAFLD) is the most common chronic liver disease and is the leading cause of liver transplants and liver-related death^1–4^. As a hepatic manifestation of obesity-related metabolic syndrome, NAFLD is tightly associated with type 2 diabetes, dyslipidemia, and cardiovascular disease^5–7^. NAFLD begins with simple steatosis due to excess accumulation of lipids in the liver. However, it can develop into a more severe subtype, nonalcoholic steatohepatitis (NASH), and can further progress to fatal cirrhosis and hepatocellular carcinoma^1,2^. Despite its striking global increase and clinical importance, the pathogenesis of NAFLD is not clearly understood, and surprisingly, there is no approved drug for treating NAFLD/NASH.

AMP-activated protein kinase (AMPK) is a master cellular energy sensor that has received much attention as a promising therapeutic target for obesity-associated metabolic disorders, including NAFLD/NASH^8–11^. AMPK is a heterotrimer complex that consists of a catalytic α-unit and two regulatory β and γ subunits^12^. Under energy-deprived conditions, AMPK is activated by phosphorylation at Thr-172 in the α-subunit. Activated AMPK increases cellular energy levels by promoting ATP-producing catabolic pathways and inhibiting ATP-consuming biosynthetic pathways^12^. A recent study utilizing liver-specific AMPK knock-out mice has shown that the loss of AMPK exaggerates diet-induced NASH pathology, particularly liver injury and hepatocellular apoptosis^13^. Since the activity of AMPK is reduced in obesity and NAFLD^9,14,15^, increasing AMPK activity has been suggested as an attractive therapeutic option for treating metabolic disorders, including NAFLD. Indeed, pharmacological activation of AMPK prevented NAFLD^16^, and liver-specific activation of AMPK protected against NAFLD/NASH in mice^17,18^. The regulation and function of the AMPK heterotrimer complex in physiology and disease^10,19^, reduced AMPK function in obesity and NAFLD^9,12,14^, and the development of AMPK activators to treat metabolic disorders^8,9,20^ have been thoroughly discussed in excellent reviews, so this review focuses on the emerging role of obesity-associated microRNAs (miRs) in regulating AMPK function.

MicroRNAs are small (19–23 nucleotides) noncoding RNAs that function as powerful posttranscriptional gene repressors^21^. Similar to protein-coding genes, miRs are transcribed by RNA polymerase II in the nucleus. After processing by the endonucleases Drosha in the nucleus and Dicer in the cytoplasm, mature miRs are loaded into an RNA-induced silencing complex (RISC) to repress the expression of genes by directly binding to the 3’ untranslated regions (3’ UTRs) of the target mRNAs^21^. MiRs have been intensively studied because of their crucial functions in diverse biological pathways, including development, differentiation, cell proliferation, and metabolism^22,23^. Remarkably, miRs are aberrantly expressed in numerous diseases, including obesity and NAFLD, and have emerged as promising therapeutic targets and/or diagnostic markers^23–25^.

AMPK function is aberrantly decreased in NAFLD/NASH, but the underlying mechanisms are not clearly understood. Emerging evidence indicates that dysregulated miRs in obesity inhibit the hepatic expression and activity of AMPK, promoting NAFLD/NASH^26–30^. In this review, we focus on recent studies that elucidate how miRs negatively impact hepatic AMPK, either directly or indirectly, and discuss the mechanisms that underlie the aberrant increase in the expression of some miR genes that can impact AMPK function in obesity and NAFLD. Furthermore, we discuss the role of miR-AMPK regulatory axes as novel potential therapeutic targets for treating NAFLD/NASH.

### Direct regulation of AMPK by miRs

**Fig. 1:**
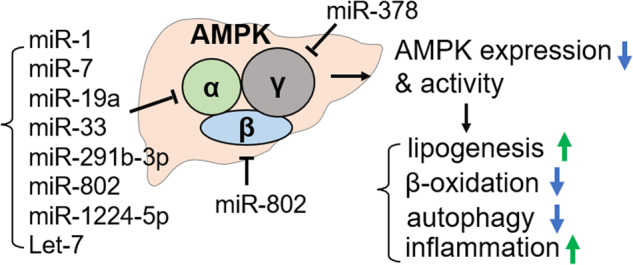
Schematic summarizing the direct repression of each AMPK subunit by miRs in the liver. Obesity-associated miRs repress hepatic expression and the activity of AMPK, resulting in increased lipogenesis, reduced mitochondrial fatty acid β-oxidation, reduced autophagy/lipophagy, and increased inflammation, which promote the development of NAFLD/NASH.

AMPK functions as an obligate heterotrimer complex that consists of α, β and γ subunits^12^. In mammals, there are two catalytic α-subunits, α1 and α2, two regulatory β-subunits, β1 and β2, and three regulatory γ-subunits, γ1, γ2, and γ3^12^. These subunits can potentially generate 12 distinct AMPK complexes with different biological functions and subcellular localizations^12,31^. Recent studies have shown that the expression of all three subunits of AMPK is inhibited by multiple miRs, leading to reduced AMPK expression and activity in obesity and NAFLD (Fig. 1). MiRs that can negatively impact AMPK function by targeting each of the AMPK subunits are listed in Table 1.

**Table 1:** List of miRs that directly repress hepatic AMPK function.

miR	Organism	Expression in obesity/NAFLD	Direct target	References
miR Let-7	Mouse	Up	AMPKα2	(Simino et al. ^43^)
miR-1	Freshwater drum	-	AMPKα1	(Chen et al. ^118^)
miR-1224-5p	Human, mouse	Up	AMPKα1	(Chen et al. ^35^)
miR-19a	Human, mouse	Up	AMPKα1	(Liu et al. ^41^)
miR-291b-3p	Mouse	Up	AMPKα1	(Meng et al. ^32^)
miR-33a/b	Human	-	AMPKα1	(Davalos et al. ^38^)
miR-378	Human, mouse	Up	AMPKγ2	(Zhang, Hu, et al. ^48^)
miR-7	Human	-	AMPKα1	(Singaravelu et al. ^116^)
miR-802	Human, mouse	Up	AMPKα1 & β1	(Sun et al. ^26^; Ni et al. ^46^)

#### Regulation of the AMPKα subunit by miRs

Numerous miRs can repress hepatic AMPK activity by directly targeting its catalytic α subunit. Hepatic expression of miR-291b-3p is elevated in both leptin receptor-deficient db/db mice and high-fat diet (HFD)-induced obese mice^32^. MiR-291b-3p directly inhibits AMPKα1, promoting hepatic lipogenesis. Downregulation of miR-291b-3p improves AMPK activity, leading to increased phosphorylation of ACC, a known target of AMPK^33,34^ that is critically involved in lipogenesis, and reduces the expression of FAS and SREBP1, key lipogenic proteins^32^.

Hepatic levels of miR-1224-5p are also elevated in both leptin-deficient ob/ob mice and dietary obese mice, and elevated levels of miR-1224-5p promote hepatic lipogenesis by directly targeting the AMPKα1 subunit^35^. Inhibition of miR-1224-5p increases AMPK protein levels, AMPK phosphorylation and subsequently phosphorylation of ACC, leading to reduced lipid accumulation in the liver.

MiR-33a and miR-33b are key posttranscriptional regulators of cellular cholesterol levels^36,37^. Recent studies have shown that these miRs are also involved in metabolic regulation through the inhibition of numerous targets, including CPT1, the AMPKα1 subunit, and IRS2^38^. Overexpression of miR-33 reduces fatty acid β-oxidation and insulin signaling by decreasing the expression of its targets, *Cpt1α, Ampkα1 and Irs2*, whereas downregulation of miR-33 has the opposite effects.

AMPK activates macroautophagy, including lipophagy, under energy-deprived conditions to maintain energy homeostasis^39,40^. MiR-19a inhibits autophagy in the liver partly by targeting AMPKα1^41^. In human patients with acute liver failure and in in vitro hepatocyte injury models, hepatic miR-19a levels are elevated, and AMPKα1 protein levels and AMPK activities are decreased, which is consistent with decreased autophagic flux^41^. Notably, miR-19a inhibits AMPK function by directly targeting the AMPKα1 subunit but also indirectly by targeting NBR2, a long noncoding RNA that acts as a positive regulator of AMPK signaling^42^.

In addition to miR-19, let-7 plays a role in reducing AMPKα2 levels, which contributes to NAFLD development^43^. Hepatic let-7 levels are elevated in newborns from obese female mice, and this elevation in let-7 levels is correlated with the levels of serum free fatty acids, glucose, and insulin in both female mice and their offspring. The overexpression of let-7 inhibits AMPK function by directly targeting the α2 subunit, and conversely, the downregulation of let-7 prevents lipid accumulation in hepatocytes^43^.

#### Regulation of the AMPKβ subunit by miRs

MiR-802 is one of the most highly upregulated miRs in the livers of obese mice and humans^44,45^. Kornfeld et al. originally reported that the overexpression of miR-802 causes glucose intolerance and insulin resistance, whereas its downregulation improves glucose regulation. Additionally, these effects are mediated partly through the silencing of a homeobox transcription factor, HNF1β (also called Tcf2)^44^.

Recently, Sun et al. reported that hepatic miR-802 levels are aberrantly elevated in the livers of NAFLD patients and obese mice and that miR-802 directly targets AMPKα and β subunits, repressing hepatic AMPK^26^. Furthermore, reduced AMPKβ1 protein levels promote the degradation of the AMPKα subunit, resulting in reduced hepatic AMPK activity^26^. Remarkably, in mice with diet-induced NASH, the overexpression of miR-802 largely abolishes the beneficial reduction in hepatic inflammation, fibrosis, and apoptosis mediated by obeticholic acid (OCA), a potent agonist of the bile acid nuclear receptor farnesoid X receptor (FXR/NR1H4). OCA is under clinical trials for the treatment of NAFLD/NASH patients^26^. Interestingly, the regulation of hepatic AMPK by miR-802 was also demonstrated in mice infected with a parasite, S. japonicum^46^. Infection of mice with S. japonicum decreases miR-802 levels and increases AMPK levels, reducing hepatic lipogenesis^46^. These recent studies showed that miR-802 directly targets the 3’UTRs of both AMPKα1 in humans and Ampkβ1 in mice^26,46^. Notably, miR-802 recognition sequences are present in the 3’UTRs of α1 and β1 transcripts in almost all mammals, suggesting that the miR-802/AMPK axes are highly conserved^26,46^.

#### Regulation of the AMPKγ subunit by miRs

NASH is the more severe form of NAFLD and is tightly linked to overnutrition, inflammation, liver injury, and decreased AMPK activity^47^. Recently, Song and colleagues demonstrated that miR-378 activates NF-κB/TNFα inflammatory signaling by directly targeting the AMPKγ2 subunit, identifying miR-378 as a potential therapeutic target for the treatment of NASH^48^. They found that miR-378 is elevated in the livers of diet-induced obese mice and NASH patients and that elevated miR-378 levels repress AMPKγ2 expression in the liver^48^. Furthermore, hepatic SIRT1 is negatively regulated by miR-378, potentially due to reduced AMPK function. This leads to increased acetylation of the p65 subunit of NF-κB and to increased TNFα levels and inflammation in the liver^48^.

### Indirect regulation of AMPK by miRs

**Fig. 2:**
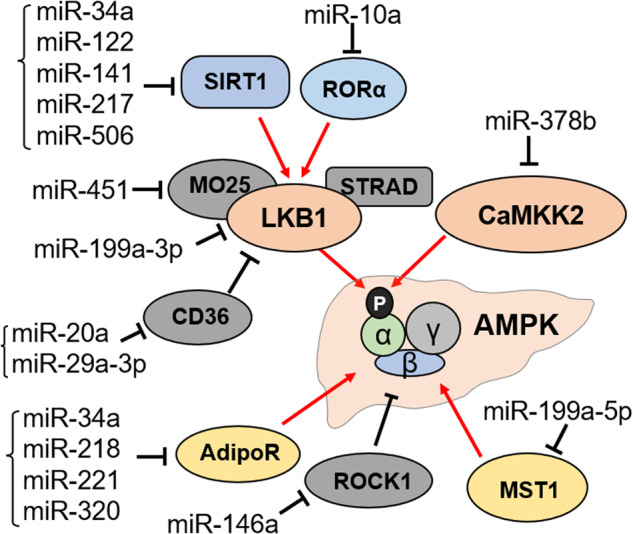
Schematic summarizing the indirect regulation of AMPK in the liver by miRs via the regulation of activators and repressors of AMPK. LKB1 and CaMKK2 are known upstream kinases of AMPK, activating AMPK via Thr-172 phosphorylation (p) in the α subunit. Red arrows indicate activation, and black blunt arrows indicate inhibition.

AMPK is a key regulator of energy metabolism, and its activity is tightly controlled by various metabolic and hormonal signals^10^. There are two well-known upstream kinases of AMPK, liver kinase B1 (LKB1) and calcium/calmodulin-dependent protein kinase kinase 2 (CaMKK2)^10,12^. Both LKB1 and CaMKK2 directly activate AMPK by phosphorylating Thr-172 in the catalytic α subunit^12^. In addition to LKB1 and CaMKK2, other cellular signaling pathways, such as adiponectin signaling, can also modulate AMPK activity. Thus, miRs could indirectly regulate hepatic AMPK function by targeting these upstream kinases and signaling pathways (Fig. 2). MiRs that can modulate AMPK indirectly by targeting AMPK activators or inhibitors are listed in Table 2.

**Table 2:** List of miRs that indirectly regulate hepatic AMPK function by regulating activators and repressors of AMPK.

miR	Organism	Expression in obesity/NAFLD	Direct target	AMPK function	References
miR-10a	Human	Up	RORα	Negative	(Horii et al. ^67^)
miR-122	Human, mouse	Up	SIRT1, CPEB1	Negative	(Jin et al. ^120^; Long et al. ^28^)
miR-1271	Human	-	CCNA1	Positive	(Chen, Zhao, et al. ^77^)
miR-130b-3p	Human, mouse	Down	ROCK1	Positive	(Guo et al. ^105^)
miR-130b-5p	Mouse	-	SIRT4	Negative	(Wang, Wang, et al. ^55^)
miR-141	Human	Up	SIRT1	Negative	(Yousefi et al. ^29^)
miR-146a	Human, mouse	Down	NAMPT, ROCK1	-	(Gong et al. ^71^; Chen, Tan, et al. ^117^)
miR-199a-3p	Human	Up	LKB1	Negative	(Lee et al. ^50^)
miR-199a-5p	Human, mouse	Up	MST1	Negative	(Li et al. ^69^)
miR-200a-3p	Human, mouse	-	SIRT1	Negative	(Wu et al. ^104^)
miR-20a-5p	Human, mouse	Down	CD36	Positive	(Wang et al. ^73^)
miR-217	Mouse	Up	SIRT1	Negative	(Yin et al. ^119^)
miR-218	Human	Up	AdipoR2	Negative	(Du et al. ^62^)
miR-22	Human, mouse	Up	FGFR1	Negative	(Hu et al. ^30^)
miR-221	Human, mouse	Up	AdipoR1	Negative	(Lustig et al. ^60^)
miR-29a	Human, mouse	-	CD36	Positive	(Lin et al. ^74^)
miR-320	Rat	Up	AdipoR1	Negative	(Wei et al. ^61^)
miR-34a	Human, mouse	Up	SIRT1, AdipoR2, NAMPT	Negative	(Lee et al. ^52^; Wen et al. ^63^; Choi et al. ^90^)
miR-378b	Human, mouse	Up	CaMKK2	Negative	(Wang, Lu, et al. ^55^)
miR-448	Human, mouse	-	MAGEA6	Positive	(Guo et al. ^76^)
miR-451	Human, mouse	Up	MO25	Negative	(Hur et al. ^27^)
miR-506-3p	Human, mouse	Up	SIRT1	Negative	(Hu et al. ^30^)
miR-519d	Human	-	Rab10	Positive	(Zhang, Pan, et al. ^75^)
miR-665-3p	Mouse	Up	FNDC5	Negative	(Yu et al. ^68^)

#### Indirect repression of AMPK via a miR-LKB1 axis

LKB1 directly regulates Thr-172 phosphorylation in the AMPKα subunit, promoting its activation^49^. Previous studies in several genetic mouse models revealed that LKB1 downregulation severely impairs AMPK activation in most tissues, including hepatic tissue^49^. MiR-199a-3p has been shown to indirectly reduce AMPK activity by targeting LKB1. Hepatic expression of the bile acid nuclear receptor FXR is low in fibrotic livers of mice and humans, which is associated with the upregulation of FXR-repressed miRs, including miR-199a-3p^50^. MiR-199a-3p directly targets LKB1, reducing AMPK activity in the fibrotic liver. Treatment with FXR agonists, both in vitro and in vivo, increased LKB1 expression and downstream p-AMPK levels, protecting hepatocytes from injury in an AMPK-dependent manner^50^.

Calcium-binding protein 39 (CAB39, also known as MO25) is necessary for the kinase activity of LKB1^49^. Hur et al. reported that CAB39 is a target of miR-451 and that reduced miR-451 levels result in increased LKB1/AMPK activity. This causes AKT activation, NF-κB nuclear translocation, and increased IL-8 production, which is consistent with higher serum IL-8 and TNFα levels in NASH patients^27^.

SIRT1 deacetylase is a well-known positive modulator of LKB1^51^. SIRT1 indirectly activates AMPK in part via the deacetylation of LKB1, influencing its localization and activity^51^. Several studies have revealed that miRs regulate AMPK function through the SIRT1-LKB1 axis. In NAFLD patients and mouse models, elevated miR-34a, miR-122, miR141 and miR-506-3p levels were reported to promote hepatic steatosis by targeting SIRT1, causing decreased LKB1/AMPK function in the liver^28,29,52,53^.

#### Indirect repression of AMPK via a miR-CaMKK2 axis

CaMKK2 is also a known upstream kinase of AMPK^10,12^. CaMKK2 promotes gluconeogenesis and suppresses lipogenesis in the liver in part by regulating AMPK activity^54^. MiR-378b is induced by ethanol treatment in both mouse and human hepatocyte cell lines and promotes hepatic lipid accumulation by directly targeting CaMKK2^55^. The elevation in miR-378b levels results in reduced CaMKK2 expression, thereby reducing p-AMPK and p-ACC levels and causing increased lipid accumulation in vitro and in vivo. Conversely, inhibition of miR-378b protects mice against ethanol-induced hepatic steatosis^55^.

#### Indirect repression of AMPK via a miR-AdipoR axis

Adiponectin is an adipocyte-derived hormone that plays an important role in metabolic regulation. In metabolic tissues, including liver and skeletal muscle tissue, activation of adiponectin receptors promotes the activation of AMPK, increasing fatty acid β-oxidation and glucose utilization^56,57^. Conversely, the downregulation of adiponectin receptors (AdipoR), either AdipoR1 or AdipoR2, impairs AMPK activity, promoting liver fibrosis and metabolic disorders^58,59^.

MiR-221 directly targets AdipoR1, resulting in impaired adiponectin signaling and repressed AMPK activity in liver and muscle^60^. MiR-320 also targets AdipoR1^61^. After postduodenal-jejunal bypass (DJB) surgery, miR-320 levels were significantly decreased in the livers of rats. This resulted in an increase in AdipoR1 levels, causing an elevation in p-AMPK levels^61^.

Downregulation of AdipoR2 by miRs was also shown to reduce AMPK function. MiR-218 reduced AMPK function by targeting AdipoR2 in HepG2 cells, resulting in reduced adiponectin sensitivity and reduced AMPK activity^62^. Obesity promoted by resistin is mediated in part by elevations in the levels of miR-34a, which directly targets AdipoR2^63^. Inhibition of AdipoR2 by miR-34a provides a possible mechanism by which resistin affects fatty acid oxidation and mitochondrial biogenesis^63^.

#### Repression of AMPK via miR inhibition of additional AMPK activators

Retinoic acid receptor-related orphan receptor α (RORα/NR1F1) plays an important role in the maintenance of hepatic lipid homeostasis^64–66^. Lee and colleagues originally reported that RORα attenuates hepatic steatosis by activating AMPK^65^. RORα modulates the hepatic expression of numerous genes involved in hepatic lipid metabolism, such as lipogenesis and mitochondrial β-oxidation genes, in part by promoting AMPK activity^65^. Remarkably, liver-specific deletion of *Rorα* aggravates diet-induced NASH in mice by inducing mitochondrial dysfunction^64^ and promotes obesity, hepatic steatosis, and insulin resistance by activating PPARγ^66^. Regarding RORα function, elevated levels of miR-10 in chronic hepatitis C was shown to directly repress the expression of RORα, which downregulates the expression of various RORα-regulated genes and the levels of phosphorylated AMPK in hepatocytes^67^.

Yu et al. reported that hepatic miR-665-3p levels are increased in mice fed a HFD and that elevated miR-665-3p levels directly target FNDC5, reducing AMPK activity and promoting NAFLD^68^. Mammalian sterile 20-like kinase 1 (MST1) is a key component of the Hippo signaling pathway. Li et al. showed that an adipocyte-derived miR, miR-199a-5p, reduces hepatic expression of MST1 and downregulates the downstream Hippo pathway, including AMPK, promoting hepatic lipid accumulation^69^.

Chronic metabolic disorders, including obesity-associated metabolic diseases, are tightly linked to aging^70^. Recently, Gong et al. reported that an aging-associated miR, miR-146a, impedes the antiaging effect of AMPK in part by targeting NAMPT, a key enzyme in NAD^+^ synthesis, and subsequently inhibiting the NAD^+^-dependent SIRT1 deacetylase, a positive modulator of AMPK^71^.

#### Activation of AMPK via miR inhibition of AMPK repressors

Although many miRs negatively impact AMPK, some miRs can promote AMPK activity indirectly by targeting inhibitors of AMPK. For example, CD36 negatively regulates AMPK by regulating LKB1^72^. Both miR-20a-5p and miR29a directly target CD36 and ameliorate NAFLD, potentially by increasing AMPK activity^73,74^. MiR-519d directly targets Rab10 in hepatocellular carcinoma (HCC) tissues and cell lines, and overexpression of miR-519d induces autophagy and apoptosis by increasing AMPK activity in a Rab10-dependent manner^75^. MiR-448 promotes AMPK in HCC by repressing melanoma antigen gene-A6 (MAGEA6) to inhibit cancer cell self-renewal^76^. Furthermore, miR-1271 targets cyclin A1. The overexpression of miR-1271 increases AMPK activation, reducing cell migration and promoting HCC apoptosis^77^. However, further studies will be needed to determine whether these miRs that affect AMPK activity in HCC have a role in NAFLD/NASH.

#### Role of exosomal miRs in the development of NAFLD via cell‒cell communication

Mounting evidence indicates that miRs are major components of exosome extracellular vesicles^78–80^, which play a critical role in various cellular processes by mediating cell‒cell communication^81,82^. Exosomal miRs are being tested as potential therapeutic tools and biomarkers for many human diseases, including chronic liver diseases such as NAFLD and cancer^78,79,81^. In this review, we focused on miRs generated in hepatocytes that can impact the development of NAFLD/NASH, particularly miRs targeting AMPK. However, exosomal miRs derived from other liver cells, such as Kupffer cells and liver-resident macrophages, are also critically involved in the pathogenesis of chronic liver diseases, including NAFLD/NASH^80–82^. Recently, Gao et al. showed that exosomal miR-690 derived from Kupffer cells directly targets NAD^+^ kinase, inhibiting lipogenesis in hepatocytes, inflammation in Kupffer cells, and fibrosis in stellate cells^83^. Remarkably, the miR-690 levels in Kupffer cells were low during NASH development, and miR-690 treatment resulted in beneficial therapeutic effects, such as decreased fibrosis and steatosis and restored Kupffer cell function in NASH mice^83^. Exosomal miR-500 derived from lipopolysaccharide-activated macrophages was also shown to promote stellate cell proliferation and activation, promoting liver fibrosis^84^. It will be interesting to determine whether hepatic AMPK function is altered by exosomal miRs during NASH development through liver cell‒cell interactions.

### Mechanisms underlying the aberrant expression of *miRs* in obesity and NAFLD

The expression of RNA polymerase II-expressed genes, including miR genes, is regulated by various transcription factors under the control of different cellular signaling pathways^21^. Understanding the mechanisms by which miR gene expression is altered in obesity and NAFLD may provide new insights into the development of therapeutic agents. In this review, we discuss the regulation of the expression of *miR-802, miR-34a, and miR-378*, which negatively impact hepatic AMPK function.

#### Aberrant upregulation of miR-802

**Fig. 3:**
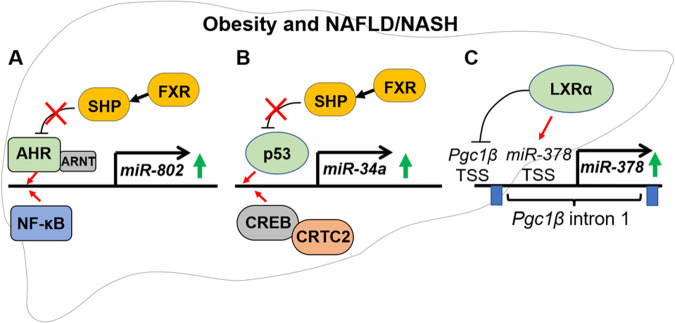
Underlying mechanisms of the aberrant expression of *miR-802, miR-34a, and miR-378*, which can negatively impact AMPK function in obesity and NAFLD/NASH. **A** Hepatic *miR-802* expression is elevated due to an impaired FXR/SHP nuclear receptor cascade and increased NF-kB inflammation signaling. **B** Hepatic m*iR-34a* expression is elevated due to impaired FXR/SHP function and obesity-induced CRTC2, a transcriptional coactivator of CREB. **C** Hepatic *miR-378* expression is increased partly due to nuclear receptor LXRα activity. LXRα promotes the transcription of *miR-378* embedded within *Pgc1β* intron 1 but inhibits the transcription of the *Pgc1β* gene. Red arrows indicate activation, and black blunt arrows indicate inhibition.

Numerous miRs are aberrantly expressed in obesity, and these alterations contribute to obesity-associated metabolic problems^85,86^. For example, defective function of nuclear receptors, such as FXR and small heterodimer partner (SHP/NR0B2), an FXR-induced orphan nuclear receptor, contributes to the aberrant expression of miRs in obesity^87^. Global small RNA-seq analysis in mice with liver-specific downregulation of SHP expression revealed that FXR-induced SHP inhibits the hepatic expression of many miRs that are involved in metabolic regulation, such as miR-802 and miR-34a^87^. Notably, miR-802 was reported to be one of the most highly upregulated miRs in overweight patients and diet-induced obese mice^44,45^, where SHP nuclear localization and its gene-regulatory function are compromised^45,87^. Under physiological conditions, SHP inhibits the activity of the aromatic hydrocarbon receptor (AHR), a transcription activator of *miR-802*, leading to the repression of *miR-802*^45^. However, in obesity and NAFLD, a defective FXR-SHP cascade results in increased AHR occupancy at the *miR-802* promoter and increased *miR-802* gene expression (Fig. 3A). In addition, NF-κB signaling promotes miR-802 expression in the liver, which is consistent with findings that there are multiple binding sites for NF-κB and STATs in the promoter region of *miR-802* (Fig. 3A)^26,46^.

Gene expression of *miR-802* can also be upregulated by other transcription factors in a tissue-specific manner. Recently, Zhang et al. demonstrated that miR-802 levels are elevated in the pancreatic islets of obese mouse models and identified FOXO1 as a transcriptional activator of *miR-802*^88^. They further showed that elevated levels of *miR-802* repressed its targets, NeuroD1 and Fzd5, leading to impaired Ca^2^ signaling and the inhibition of insulin gene transcription and secretion^88^.

#### Aberrant upregulation of miR-34a

MiR-34a is also an obesity-induced miR that can indirectly repress hepatic AMPK by inhibiting activators of AMPK, including SIRT1, NAMPT, and PPARα^52,89,90^. Hepatic expression of *miR-34a* is regulated by different transcription factors and mechanisms. For example, the tumor suppressor p53 is a well-known transcriptional activator of *miR-34a*^91,92^. In a positive feedback loop, p53 upregulates *miR-34a*^93,94^, and in turn, p53-induced miR-34a increases p53 function partly by repressing several targets that inhibit p53, such as SIRT1^52^. SHP normally inhibits the expression of *miR-34a* partly by blocking p53 activity so that defective SHP function in obesity and NAFLD unlocks the positive feedback loop. This leads to an aberrant elevation in miR-34a levels, causing further progression of metabolic diseases (Fig. 3B)^52^. In addition, the negative correlation between miR-34a expression and the methylation level of the CpG island in the miR-34a promoter suggests a role of DNA methylation in *miR-34a* expression^95^.

Recent studies have also shown that *miR-34a* is upregulated by cAMP response element-binding protein (CREB) and its transcriptional coactivator, CREB-regulated transcriptional coactivator 2 (CRTC2), which are key mediators of cAMP/PKA signaling-induced transcriptional events^96^. Utilizing CRTC2 liver-specific knockout mice, Koo and colleagues demonstrated that CRTC2 induces the hepatic expression of *miR-34a*. This leads to the repression of *Fgf21*, a key metabolic hormone that lowers lipid levels and sensitizes insulin action by targeting the Sirt1/Pparα/Fgf21 axis^97^. Furthermore, HFD-induced activation of CRTC2 increases miR-34a/mTOR activity in the liver, promoting NAFLD via the induction of lipogenesis and the inhibition of lipophagy^98^ (Fig. 3B). Remarkably, there is a strong association between CRTC2 activity and miR-34a/mTORC1 in NAFLD patients, indicating a conserved role of CRTC2 in promoting NAFLD among species^98^.

#### Aberrant upregulation of miR-378

MiR-378 is involved in numerous metabolic pathways partly by inhibiting AMPK function by targeting the AMPKγ subunit^48,99^. *MiR-378* is embedded in intron 1 of *Pgc1β* and counterbalances the metabolic actions of PGC1β, a transcriptional coactivator that regulates mitochondrial biogenesis and fatty acid metabolism^100,101^. Hepatic miR-378 levels are upregulated in HFD-fed mice and NAFLD patients^100,101^. Although *miR‐378* is embedded within the intron of *Pgc1β*, in recent studies, Song and colleagues have shown that miR‐378 possesses its own promoter and that its transcription is independent of the host *Pgc1β* gene^101^. They identified a transcription start site (TSS) of *miR-378*. They further found that the nuclear receptor liver X receptor alpha (LXRα) activates the transcription of *miR-378* but inhibits the transcription of the *Pgc1β* gene^101^ (Fig. 3c). These findings are consistent with the role of LXRα in promoting lipogenesis and impairing fatty acid oxidation, which contributes to the development of NAFLD^101^.

#### Regulation of miR expression via long noncoding RNAs

Long noncoding RNAs (lncRNAs) are a type of noncoding RNA that are at least 200 nt long^102^. LncRNAs have important roles in diverse biological processes and have gained increasing attention^103^. Although the role of lncRNAs in metabolic regulation is still controversial, many have been reported to act by regulating miRs. For example, TUG1 functions as a microRNA sponge that inhibits miR-200a-3p, which targets SIRT1, negatively influencing AMPK^104^. Similarly, HOTAIR binds to miR-130b-3p, reducing free miR-130b-3p levels^105^. Because miR-130b-3p targets the AMPK inhibitor ROCK1^106^, HOTAIR could promote NAFLD indirectly through the miR-130-3p/ROCK1/AMPK axis.

#### Therapeutic potential of miR-AMPK regulatory pathways

Since the activity of AMPK is reduced in obesity, increasing AMPK activity has been suggested as an attractive therapeutic option for obesity-associated metabolic diseases, including NAFLD/NASH^8–10,19,20^. For example, AMPK activation by PXL770 improved many metabolic features in patients with type 2 diabetes and NAFLD^107^. In this regard, targeting the miR-AMPK axis would be a promising strategy to treat NAFLD/NASH. A recent study has shown that miR-802 blocks the beneficial effects of OCA, a semisynthetic FXR agonist currently under clinical trials for NASH^45^. OCA treatment decreased the insulin resistance and fatty liver caused by HFD feeding, and overexpressing miR-802 largely abolished these beneficial effects^45^. A follow-up study showed that OCA significantly increased the phosphorylation levels of hepatic AMPK, reducing NASH pathologies, liver injury and apoptosis, and these OCA-mediated beneficial effects were largely abolished by the overexpression of miR-802 in dietary NASH mice^26^. Together with the numerous studies discussed above, targeting miR-AMPK pathways has been shown to be a promising approach for NAFLD/NASH.

Currently, the miR therapeutic approach, either utilizing miR mimetics to increase miR levels or utilizing miR inhibitors to block miR functions, is making great progress, and many miR-related drugs are already in clinical trials. MiR-based clinical trials and the challenges have been summarized in several excellent reviews^25,108–111^. In general, a good delivery system, good specificity with the absence of off-targeting effects, and minimal immunogenicity are desirable. Numerous studies have focused on targeting either miRs or AMPK separately, but cotreatment targeting both might be more effective with fewer side effects, consistent with the known benefits of combined treatment^112,113^. For example, in NALFD, the AMPK expression levels are low, so AMPK activators at low doses might be ineffective. Thus, the combination of low-dose miR-based agents to increase AMPK levels with low-dose AMPK activators may be synergistic.

## Conclusion and future perspectives

Aberrantly expressed miRs in obesity and NAFLD inhibit hepatic AMPK function, disrupting normal liver physiology. In this review, we summarized the recent studies of the miR-AMPK pathway, particularly in the liver, that involve either direct repression of one of the AMPK subunits or indirect regulation by targeting AMPK modulators. We also discussed how miR genes that can inhibit AMPK are aberrantly expressed in the liver. Furthermore, we discussed the therapeutic potential of targeting the miR-AMPK pathway.

Therapeutic activation of AMPK in treating obesity-associated metabolic disorders, including NAFLD/NASH, has been extensively tested with different strategies^19,107,114,115^. As our understanding of the miR-AMPK pathway expands and miR-based therapeutics evolve, miRs may function as important therapeutic regulators restoring AMPK. The current development of miR-based therapeutics is encouraging, but there are still many hurdles to overcome before an effective miR-based treatment for NAFLD can be developed. The miR-AMPK axes are still not very well characterized, and the continued development of better delivery vehicles of the miR-based agents should be possible. Nevertheless, the overall development of miR-based therapeutics is still in early stages. However, based on the great potential demonstrated in recent studies of miRs and the great efforts that have been invested in AMPK-based therapeutics, we should witness rapid growth of miR-AMPK therapeutics in the near future.
